# Conjugated Nitroolefins as Twofold Electrophiles for the Assembly of Five-Membered Monoheterocycles via MIRC [3+2] Annulation: An Update on Synthetic and Mechanistic Aspects

**DOI:** 10.3390/molecules31050803

**Published:** 2026-02-27

**Authors:** Lara Bianchi, Massimo Maccagno, Giovanni Petrillo, Cinzia Tavani

**Affiliations:** Department of Chemistry and Industrial Chemistry (DCCI), University of Genova, Via Dodecaneso 31, 16146 Genova, Italy; massimo.maccagno@unige.it (M.M.); giovanni.petrillo@unige.it (G.P.);

**Keywords:** five-membered monoheterocycles, nitroolefins, [3+2] annulations, Michael-Initiated Ring Closure (MIRC)

## Abstract

Five-membered monoheterocycles, either isolated or embedded in more complex systems, are ubiquitous structural motifs in nature and hence privileged targets of synthetic chemistry. Among a plethora of methodologies used for their assembly, [3+2] annulation strategies keep attracting particular interest among chemists, partly because of some significant characteristics from both the operative and the environmental viewpoints. Herein, the extensive use of conjugated nitroolefins as twofold electrophilic, two-carbon components of [3+2] MIRC (Michael-Initiated Ring Closure) annulations is reviewed as a practical and mechanistic update covering the last decade (2015–2025).

## 1. Introduction

Among the vast and ubiquitous class of five-membered heterocycles, those containing just one heteroatom are widespread in nature, characterized by different degrees of unsaturation, either isolated or embedded in fused polycyclic systems, and this feature effectively contributes to their biological, pharmacological/medicinal, photochemical or technological properties [1,2,3,4,5].

It is not surprising, then, that the assembly of such molecular skeletons represents an everlasting task for synthetic chemists in search of higher efficiency, wider scope and/or more sustainable “green” experimental conditions (e.g., conditions devoid of metal catalysts). Accordingly, a number of methodologies have been developed within the framework of general protocols represented, e.g., by concerted 1,3-dipolar cycloadditions (the classical, very wide-scope Huisgen access to five-membered heterocycles endowed with different types or numbers of heteroatoms and/or with a variable degree of unsaturation) [1,6] or stepwise [3+2] annulations, sometimes as part of more complex cascade processes [1].

**Scheme 1:**
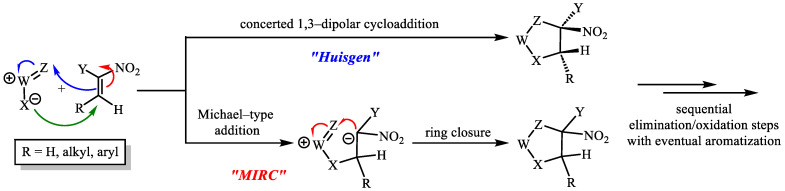
Nitroolefins as dipolarophiles in concerted (“Huisgen”) or stepwise (“MIRC”) 1,3-dipolar cycloadditions to pentatomic heterocycles.

In the current multifaceted panorama of approaches to pentatomic heterocycles, nitroolefins have long proved to be efficient two-carbon building-blocks [7], e.g., as dipolarophiles in the Huisgen reactions just cited [6,8,9,10,11,12,13,14], where the cycloaddition product retains the nitro group: this, if needed, could be lost via 1,2-elimination with aromatization (Scheme 1). A variant of the well-established concerted process is represented by a stepwise path, e.g., Barton–Zard pyrrole synthesis [15,16,17,18]. An example of the stepwise dipolar cycloaddition is sketched in Scheme 1, where an initial Michael-type addition of an allylic-type dipole to the nitroolefin is followed by ring closure (MIRC: Michael-Initiated Ring Closure [19,20,21,22]), the main difference between the two paths being the final stereochemical outcome [9], as 1,3-dipolar cycloadditions are notoriously characterized by diastereospecificity.

In the stepwise sequence of Scheme 1, the C_α_ atom of the nitroolefin behaves as a nucleophile in the ring-closing step, i.e., the intramolecular version of the textbook Michael addition, which ends up with an intermolecular coupling of C_α_ with an external electrophile (e.g., a proton) within an overall tandem addition to the double bond.

**Scheme 2:**
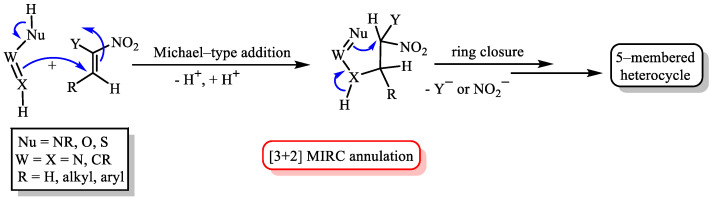
Nitroolefins as twofold electrophiles in stepwise [3+2] MIRC cycloadditions to pentatomic heterocycles.

Interestingly enough, though, following the initial nucleophilic attack to C_β_, the C_α_ atom of the nitroolefin could behave in turn as an electrophilic center: this requires, of course, the participation of the three-atom partner in the coupling process as a twofold nucleophile, as exemplified in the non-exhaustive Scheme 2.

As a matter of fact, the involvement of nitroolefins as twofold electrophiles (“latent 1,2 bis-electrophiles” [4]) for the assembly of five-membered heterocycles by means of the [3+2] MIRC strategy is well reported in the literature (see, e.g., refs. [9,13,14,23,24,25] for some significant recent reviews on different aspects relevant to the synthesis of pyrroles). The continuous application of such a strategy to different systems in different experimental conditions, coupled with our long-standing interest in the exploitation of conjugated nitrodienes as building blocks in synthetic organic chemistry [26,27,28,29,30,31,32,33,34,35,36,37,38,39,40], prompted us to update the topic. Herein, we collect and organize the latest reports relevant to the approach to five-membered monoheterocycles, either isolated or as part of fused polycyclic systems: this requires that just one of the nucleophilic functionalities of the three-atom reagent of Scheme 2 be a heteroatom [Nu = NH(R,Ar), O, S] within a Nu-C-C bidentate nucleophilic fragment such as an enamine or an enaminocarbonyl, an enol, or an enethiol.

**Scheme 3:**
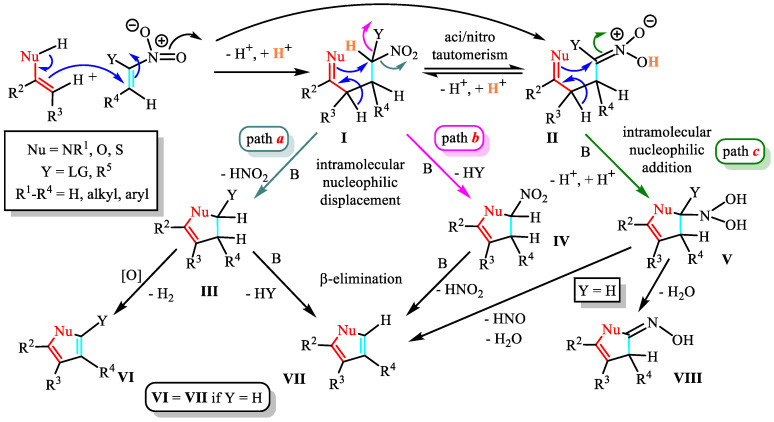
Nitroolefins as twofold electrophiles in [3+2] MIRC annulations with bidentate α,β-unsaturated nucleophiles.

Scheme 3 integrates Scheme 2 regarding the viable mechanistic pathways reported in the literature for the MIRC annulation from nitroolefins to five-membered monoheterocycles. Actually, the ring-closing intramolecular nucleophilic attack could be not only a substitution, where the leaving group would be represented by the nitro group itself (path “***a***”, leading to intermediate **III**) or by a better leaving group (Y) present at C_α_ (path “***b***”, leading to intermediate **IV**): an alternative pathway is represented by a nucleophilic *addition* onto a modified nitrogen functionality (path “***c***”) leading to a labile, not isolable intermediate (**V**) which would evolve with elimination of water and nitroxyl (HNO), an elusive species which could be confirmed by instrumental detection or by trapping of nitrogen oxides therefrom (e.g., N_2_O, generated by dehydration of hyponitrous acid, a dimer of nitroxyl) [41,42]. Some significant variants of the mechanistic paths depicted in Scheme 3 will also be encountered below in the text, essentially due to the presence, in Y, of structural motifs which would influence the course of the reaction: see, for instance, the use of Morita–Baylis–Hillman (MBH) adducts of nitroolefins [43] hereinafter.

Herein, an update on the widespread use of conjugated nitroolefins as twofold electrophilic, two-carbon components in [3+2] annulations to five-membered monoheterocycles is provided, basically covering the last decade (2015–2025).

The review is organized in sections on the basis of the nature of the heteroatom characterizing the five-membered heterocycle: nitrogen, oxygen, or sulfur. Reference to path “***a***”, “***b***” or “***c***” of Scheme 3 is made for the mechanism proposed, if any, in each citation.

## 2. Assembly of Five-Membered Monoheterocycles

### 2.1. Five-Membered Nitrogen Heterocycles

A comprehensive list of references to more or less classical methodologies addressing the ubiquitous class of pyrrole derivatives can be found in a recent publication [44]. As mentioned in the introduction, well justified by the great interest in the pyrrole nucleus, the more specific topic of the synthesis of pyrroles from nitroolefins has been dealt with in a number of recent comprehensive reports. In this regard we should mention a 2016 short review covering the 2013–2015 literature [23], a 2020 review updated to 2018 [9] and a very recent 2025 review covering the literature up to 2022 [24]; furthermore, the application of β-nitrostyrenes, in particular, as substrates has been reviewed in 2023, covering the 2014–2022 literature (also providing an extensive list of references to the preparation of β-nitrostyrenes) [25] and in a 2020 review [45], more specifically dealing with multicomponent reactions. For the sake of completeness some pertinent reports from such reviews will be cited hereinafter.

An ever-increasing number of syntheses of pyrroles [44,45,46] take advantage of the benefits offered by multicomponent (three- or four-component), one-pot domino reactions (MCRs), a strategy more and more popular in the fields of organic synthesis, as it offers significant benefits from a practical point of view: among them, the possibility of generating the substrates for the construction of the target molecule starting from easily available, simpler precursors, while being generally gratified by excellent yields [47,48,49]. Within the more specific topic herein, the protocol allows one, e.g., to assemble enaminoketones or enaminoesters (providing the N-C-C fragment of the pyrrole nucleus) by coupling between 1,3-dicarbonyls and amines, while nitroolefins can be generated by means of the Henry condensation between nitroalkanes and aldehydes or ketones.

**Scheme 4:**
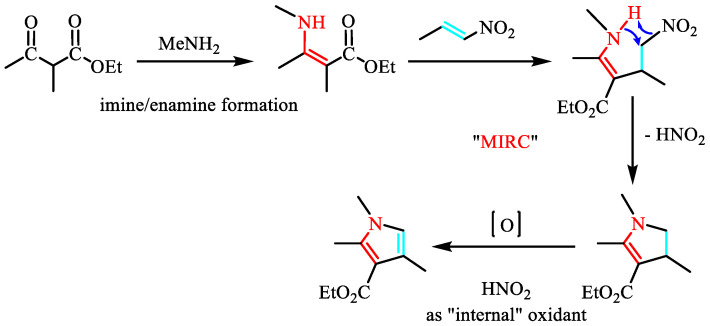
The pioneering three-component domino pyrrole synthesis by Grob and Camenisch.

The topic of MCR processes in the synthesis of pyrroles from b-nitrostyrenes has been extensively discussed in a 2020 [45] and a 2024 review [44], while the pioneering three-component domino Grob–Camenisch approach (Scheme 4) [50] from a nitroolefin, a 1,3-dicarbonyl compound and an amine has been the subject of a 2025 review [24] covering the literature up to 2022; therefore, with some exceptions selected in order to provide better coverage of the subject, only the MCR literature of the last decade not reported in the cited reviews will be reported herein.

On the other hand, this review does not cover those pyrrole syntheses where the nitrogen atom of the final heterocycle is provided by the nitroolefin itself after reduction (cf. ref. [51] for a recent report and ref. [52] for a survey of recent examples of relevant methodologies).

#### 2.1.1. Isolated Pyrrole Rings

**Scheme 5:**
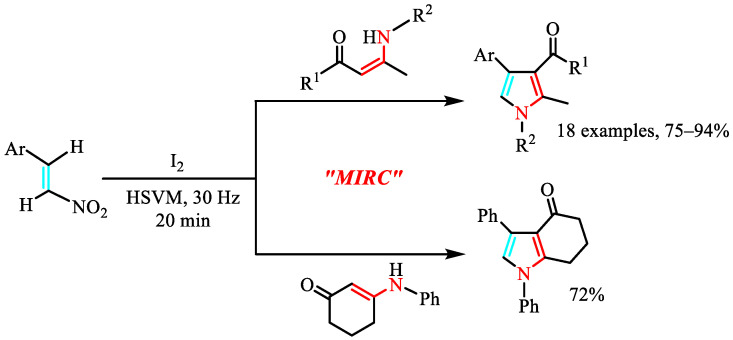
Pyrroles from nitrostyrenes and enaminoketones in HSVM conditions.

An improved pyrrole synthesis of wide scope and high yields from nitrostyrenes and enaminoketones has been reported (Scheme 5): the use of I_2_ under High-Speed Vibration Milling (HSVM) overcomes drawbacks (long reaction times, high temperatures, use of organic solvents, and poor yields) which in certain ways limit the scope and/or robustness of previous reports in a variety of experimental conditions [53].

**Scheme 6:**
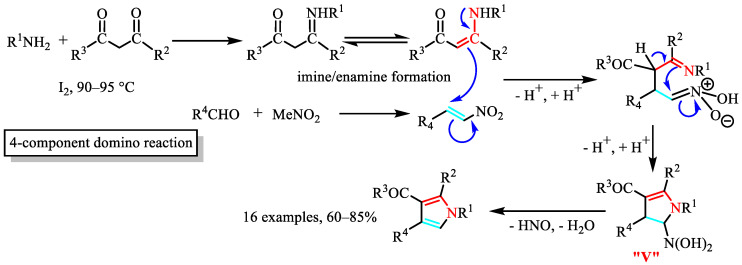
Proposed mechanism (path “***c***” of Scheme 3) for I_2_-catalyzed pyrrole synthesis via a four-component domino reaction through enaminoketones and nitroalkenes.

A MIRC process is clearly operative, initiated by attack of the enaminic C-atom to C_β_ of the nitroolefin and, although the authors do not provide any mechanistic hint, it is worth mentioning that in a previous report on a four-component access to pyrroles through enaminoketones and nitroolefins in the presence of I_2_ the intermediacy of **V** within path “***c***” of Scheme 3 was suggested (Scheme 6) [54].

**Scheme 7:**
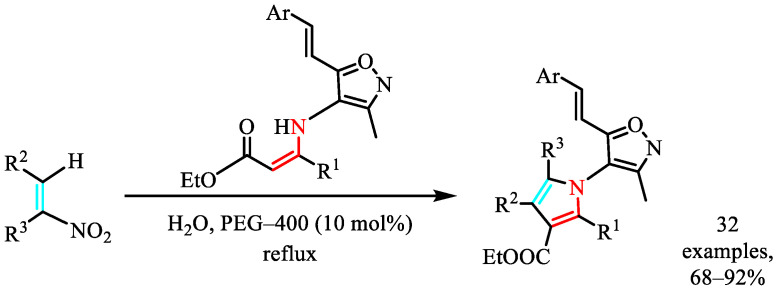
Pyrroles from nitrostyrenes and enaminoesters in H_2_O/PEG-400.

Enaminoesters have been successfully employed as twofold nucleophiles in an aqueous medium in the presence of PEG-400 (Scheme 7); the process proves to be quite effective regarding substrate scope and yields, besides being metal-free and allowing recycling of the catalyst [55].

**Scheme 8:**
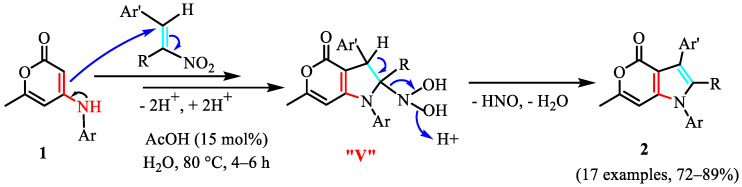
Pyrano [4,3-*b*]pyrrol-4(1*H*)-ones from nitrostyrenes and enaminolactones in H_2_O/AcOH.

Within a long-standing project on the synthesis of heterocycles, Zhang et al. published an efficient approach to obtain 2,6-dimethyl-1,3-diarylpyrano [4,3-*b*]pyrrol-4(1*H*)-one derivatives **2** from the readily available 6-methyl-4-(phenylamino)-2*H*-pyran-2-one (**1**) and nitrostyrenes in water (Scheme 8) [56]. The reaction is driven by the Michael-type attack of the enaminolactone to C_β_ of the nitrostyrene, and path “***c***” of Scheme 3 through **V** has been proposed for the MIRC process.

**Scheme 9:**
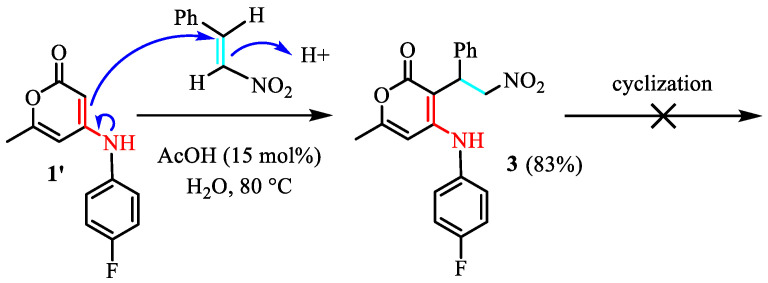
Failed ring closure after Michael-type addition.

For the reaction, despite the mild, green and environmentally benign conditions, high catalytic efficiency, and an easy work-up and purification procedure, some limitations in the substrate scope have been reported. In particular, unexpectedly enough, in the case of the 4-fluorophenyl derivative **1**′, the isolation of the non-cyclized Michael addition product **3** in an 83% yield (Scheme 9) was tentatively attributed to its high stability, thus preventing the elimination of the nitro group.

**Scheme 10:**
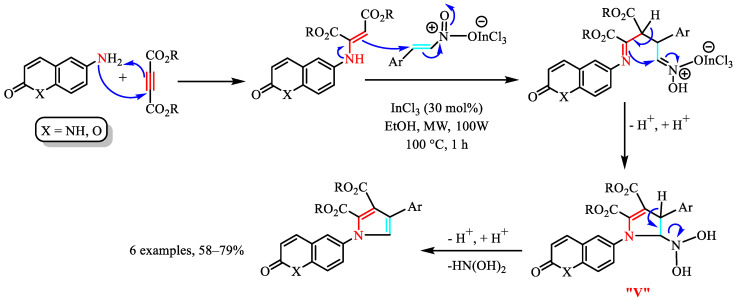
MW-assisted MCR synthesis of pyrrolocoumarin/quinolone derivatives, catalyzed by a reusable In(III) catalyst.

In the field of the MW-assisted synthesis of nitrogen heterocycles [57], a three-component MIRC access to allegedly unprecedented pyrrolocoumarin/quinolone derivatives, catalyzed by In(III), was reported in 2018 [58] (Scheme 10). Path “***c***” is proposed by the authors.

As exemplified in Scheme 10, despite the well-acknowledged environmental drawbacks associated with the use of metal catalysis, this practice has found a significant number of applications also in the field of the synthesis of pyrroles from nitroolefins; the possibility of effectively recycling the catalyst limits, though, the impact on the environment and ensures more eco-friendly conditions. More recent examples of the use of heterogeneous catalysis for the synthesis of pyrroles from nitroolefins in different experimental conditions are reported hereinafter.

**Scheme 11:**
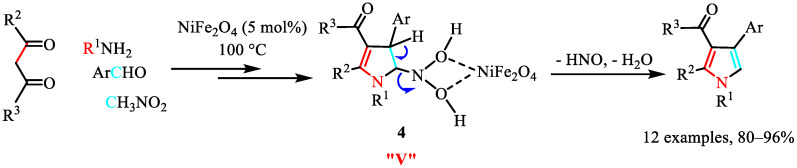
One-pot, four-component MCR synthesis of pyrroles catalyzed by NiFe_2_O_4_ nanoparticles.

A complexing effect of a metal catalyst was proposed in 2015 for the synthesis of pyrroles in the presence of NiFe_2_O_4_ nanoparticles, chosen among a few tested catalytic systems [59] (Scheme 11). The complex **4** was proposed as the key intermediate, again according to path “***c***” of Scheme 3.

**Scheme 12:**
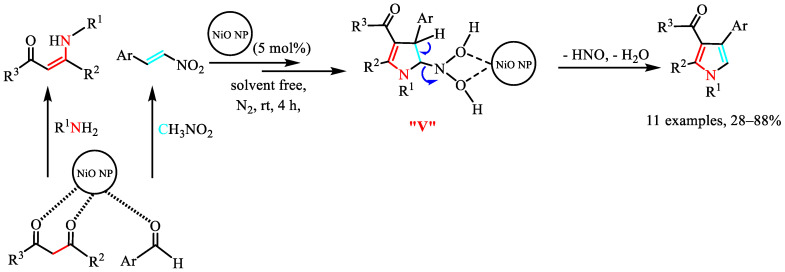
One-pot, four-component MCR synthesis of pentasubstituted pyrroles catalyzed by NiO nanoparticles.

A similar catalytic complexation was reported in 2016 [60] with the use of NiO nanoparticles (NiO NPs) within a solvent-free four-component process (Scheme 12). The proposed mechanism lines up with path “***c***” of Scheme 3, allowing the best catalytic effectiveness through complexation with the nitrogen functionality. The catalyst compares well in terms of efficacy with previously reported ones.

In 2018 the same authors developed a different heterogeneous catalytic system using Cu_2_O/Ag nanocomposites (NPs) for the synthesis of 1,2,3,4-tetrasubstituted pyrroles again via a four-component process [61]. Moderate to excellent yields and good reusability of the catalyst are positive aspects of the system.

**Scheme 13:**
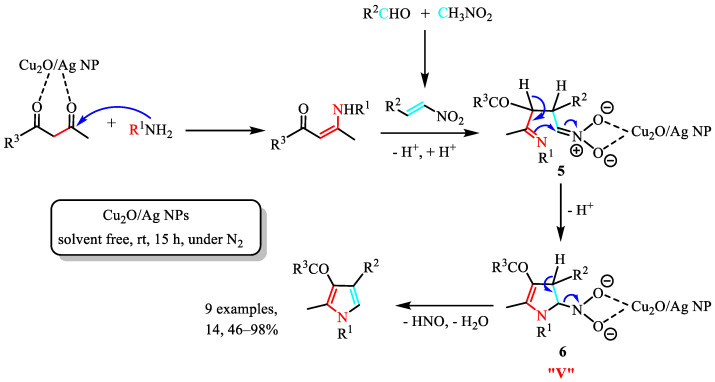
One-pot, four-component synthesis of tetrasubstituted pyrroles catalyzed by Cu_2_O/Ag nanoparticles at room temperature under nitrogen for 15 h.

Within the proposed mechanism (cf. path “***c***” of Scheme 3), the authors assign to the NPs a stabilizing/activating role of both the 1,3-dicarbonyl component and the nitro functionality in intermediates **5** and **6** (Scheme 13).

**Scheme 14:**
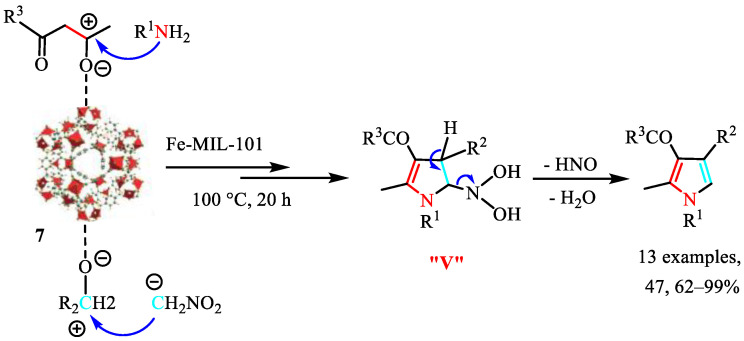
One-pot, four-component synthesis of polysubstituted pyrroles: the proposed role of the heterogeneous catalysis by highly porous Fe-MOF (Fe-MIL-101).

Interestingly enough, in the field of heterogeneous catalysis within the well-established protocol via a one-pot four-component coupling of 1,3-dicarbonyl compounds, amines, aldehydes and nitroalkanes, a 2015 report [62] assigned to an Fe-MOF (Metal–Organic Framework) catalyst a different role with respect to the one advanced in the schemes above. Thus, in the proposed mechanism the catalyst favors the two preliminary couplings between amine/dicarbonyl and nitroalkane/aldehyde in a single intermediate complex (**7**, Scheme 14).

**Scheme 15:**
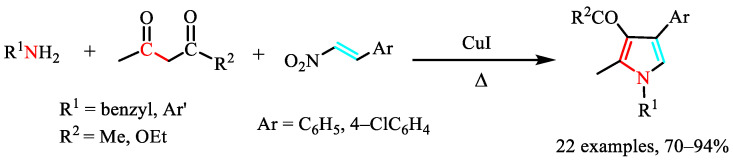
Tetrasubstituted pyrroles through a Grob–Camenisch-like Cu(I)-catalyzed, one-pot three-component reaction.

In a 2024 report, tetrasubstituted pyrroles were synthesized through a Grob–Camenisch one-pot, three-component reaction involving 1,3-dicarbonyl compounds, benzyl or aryl amines, and β-nitrostyrenes in the presence of copper catalysts (Scheme 15) [44].

According to the authors, the choice of Cu(I) among different copper catalysts tested allows the protocol to overcome limitations of previous reports, such as harsh reaction conditions, long reaction times, expensive chemicals, complex catalysts, toxic organic solvents, low atom economy, and tedious work-up and purification processes.

**Scheme 16:**
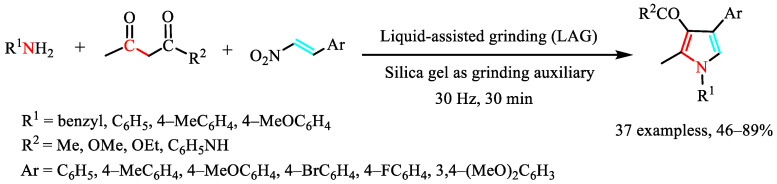
Tetrasubstituted pyrroles through a LAG-mediated MCR process.

As part of the development of green synthetic methods, in 2022 an easy one-pot, three-component synthesis of polysubstituted pyrroles was reported (Scheme 16) as a result of a catalyst-free, liquid-assisted grinding (LAG)-mediated process in the presence of methanol [63].

**Scheme 17:**
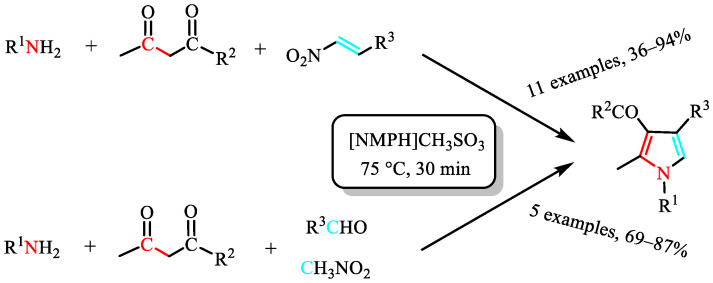
MCR synthesis of polysubstituted pyrroles in an ionic liquid: a comparison of a three-component and a four-component process.

In order to avoid metal catalysis and reach more eco-friendly conditions, the use of ionic liquids has also been applied in a 2019 one-pot MCR synthesis of tetrasubstituted pyrroles [64]. A three-component and a four-component process (Scheme 17) using *N*-methyl-2-pyrrolidonium methyl sulfonate ([NMPH]CH_3_SO_3_) as a catalyst in metal-free, optimized conditions were compared: both processes provided products in moderate to excellent yields with high atom economy.

**Scheme 18:**
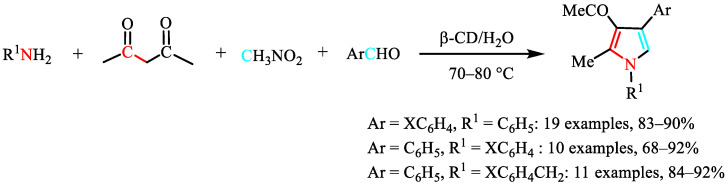
β-Cyclodextrin-catalyzed four-component synthesis of polyfunctionalized pyrroles.

Very satisfactory results gratify a β-cyclodextrin-catalyzed four-component MCR process in a 2016 report (Scheme 18) [65].

**Scheme 19:**
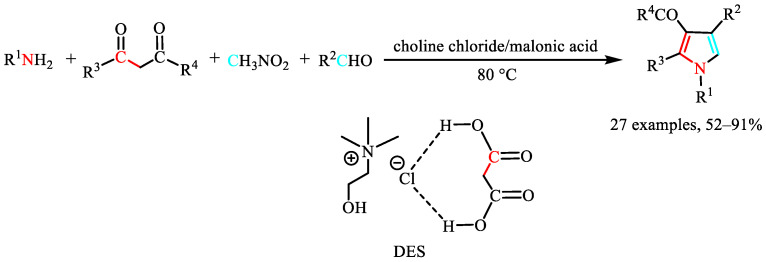
A DES-catalyzed, four-component synthesis of polyfunctionalized pyrroles.

Further recent efforts to find more and more effective catalysts in four-component protocols for polysubstituted pyrroles encompass, e.g., the use of a deep eutectic solvent (DES), prepared from choline chloride and malonic acid, with the dual role of catalyst and reaction medium (Scheme 19) [66]. The best solvent composition was selected by means of a preliminary screening, and the pyrrole yields compare very well with those from previous reports in different conditions. In particular, the authors highlight the good result for 4-nitroaniline (52%, with R^2^ = Ph, R^3^ = R^4^ = Me), a notoriously reluctant reagent.

Within path “***c***” of Scheme 3, the proposed main catalytic role of the solvent is an intermolecular H-bond activation of the aldehydic and diketone (or ketoester) carbonyls in the coupling with nitromethane and amine, respectively.

#### 2.1.2. Fused Pyrrole Rings

**Scheme 20:**
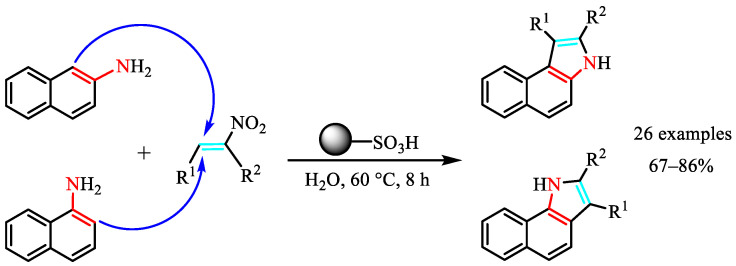
Sulfonic acid-catalyzed synthesis of benzoindoles from naphthylamines and nitroolefins.

An unprecedented, simple and very efficient protocol for the synthesis of benzoindoles and naphthofurans (see Section 2.2.2, below) is represented by the cyclization of naphthylamines and nitroolefins in water at 60 °C, catalyzed by an organic sulfonic acid (Scheme 20) [67]. Through an appropriate choice of nitroolefin, the methodology allows access to benzoindoles bearing miscellaneous substituents on the fused pyrrole ring.

**Scheme 21:**
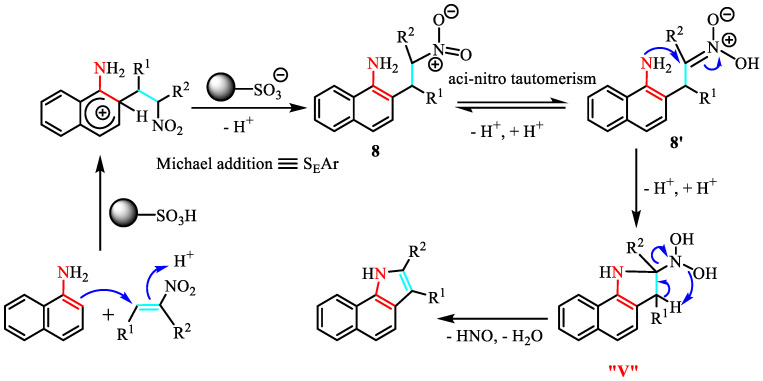
Proposed mechanism for the synthesis of benzoindoles from naphthylamines and nitroolefins.

**Scheme 22:**
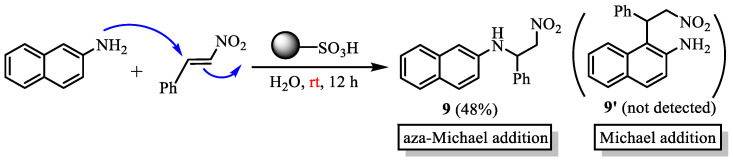
Role of the temperature in the chemoselectivity in the coupling between β-naphthylamine and β-nitrostyrene.

The product regiochemistry clearly suggests a Michael-type initial attack of the enaminic C-atom of the naphthylamine to C_β_ of the nitroalkene, the authors favoring path “***c***” of Scheme 3 as the most plausible mechanism, as exemplified in Scheme 21. The role of the temperature is surely remarkable, insofar as the model reaction between β-naphthylamine and nitrostyrene at room temperature exclusively leads to the aza-Michael addition product **9** (Scheme 22).

It should be remarked that the initial formation of intermediate **8** or **8**′ could well be regarded as an electrophilic aromatic substitution onto an activated naphthalene ring.

**Scheme 23:**
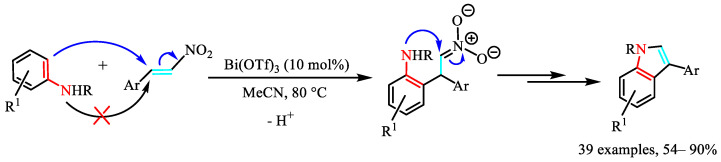
Bi(OTf)_3_-catalyzed synthesis of indoles from anilines and β-nitrostyrenes.

A regioselective synthesis of *N*-protected indoles has also been reported by means of a bismuth(III)triflate-catalyzed coupling between arylamines and trans-*β*-nitrostyrenes, where proper substituents on the aniline ring enhance its reactivity as an initial Michael C-nucleophile towards the nitrostyrene (Scheme 23) [68]. No evidence was found of an alternative aza-Michael process. The proposed mechanism is thus basically the one described in Scheme 21: an electrophilic aromatic substitution followed by ring closure through path “***c***” of Scheme 3.

**Scheme 24:**
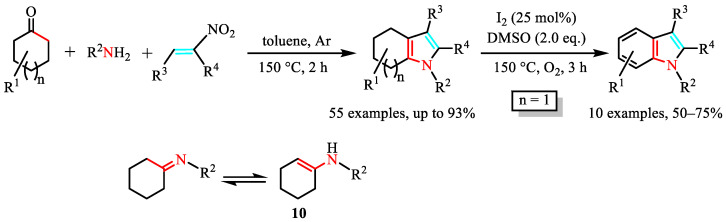
Metal-free, three-component synthesis of fused pyrroles and indoles.

A more recent contribution describes a metal-free, three-component access from amines, ketones, and β-nitrostyrenes to fused pyrroles, which could be successively effectively aromatized to substituted indoles (Scheme 24) [69]. Mechanistically, the intermediate enamine **10** performs the MIRC ring closure onto the nitrostyrene, again following path “***c***” of Scheme 3.

**Scheme 25:**
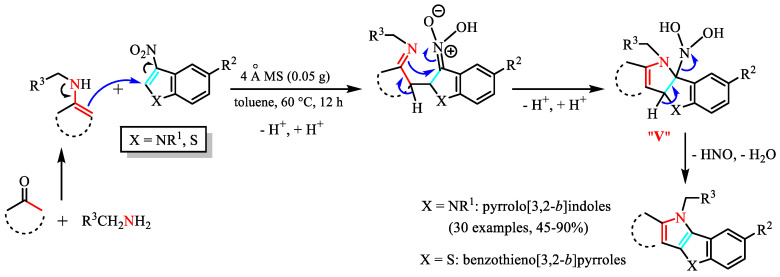
Pyrrole-fused polyheterocycles obtained from 3-nitroindoles or 3-nitrobenzo[*b*]thiophenes through a three-component process.

Interesting *N*-protected, pyrrole-fused polyheterocycles were synthesized in 2017 through a three-component annulation process from amines, ketones, and 3-nitroindoles or 3-nitrobenzo[*b*]thiophenes as twofold electrophilic nitrovinyl moieties, in the presence of 4 Å molecular sieves (Scheme 25) [70].

**Scheme 26:**
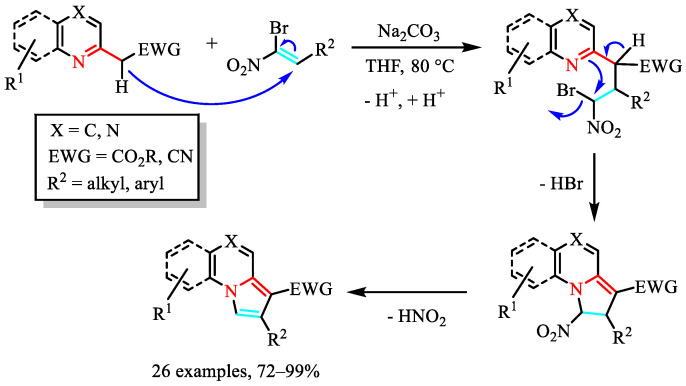
Indolizines obtained from *gem*-bromonitroolefins and 2-(methoxycarbonyl)methyl or 2-cyanomethyl-azaarenes in transition-metal-free conditions.

More recently, indolizines have been successfully obtained from 2-alkylazaarenes by exploiting the twofold electrophilicity of *gem*-bromonitroolefins, also thanks to the good leaving group ability of bromine. The cascade (*i*) Michael addition to C_β_ of the nitrostyrene and (*ii*) intramolecular S_N_2 displacement of bromine lead to a fused dihydropyrrole ring easily aromatized by a final HNO_2_ α,β-elimination. The heteroring assembly follows path “***b***” of Scheme 3 (Scheme 26) [71]. The reaction can be scaled up, and the final products are amenable to further transformations.

**Scheme 27:**
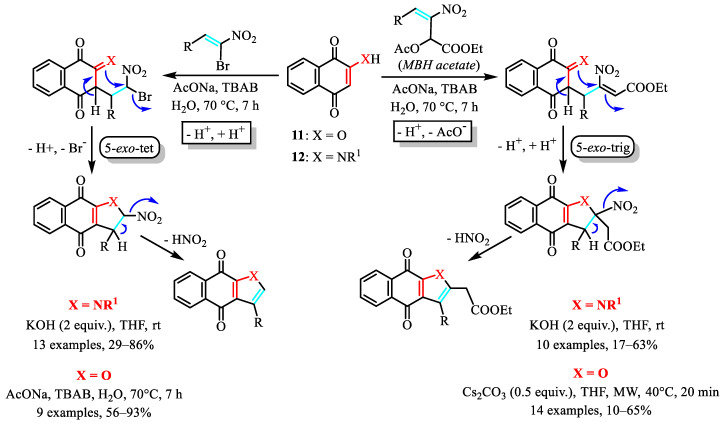
Pyrrole- or furan-fused naphthoquinones: proposed mechanisms.

Pyrrole- and furan-fused naphthoquinones with foreseeable pharmacological activity have been synthesized by reaction of lawsone (**11**) or 2-aminonaphthoquinone (**12**) with *gem*-bromonitroalkenes or nitroallylic acetates (i.e., MBH adducts of nitroolefins) (Scheme 27) [72,73]. As already pointed out, the participation of MBH adducts of nitroolefins introduces a variant in the mechanistic panorama of Scheme 3, as the ring-closing nucleophilic attack is an *addition* to a newly formed double bond at C_α_ of the nitroolefin.

**Scheme 28:**
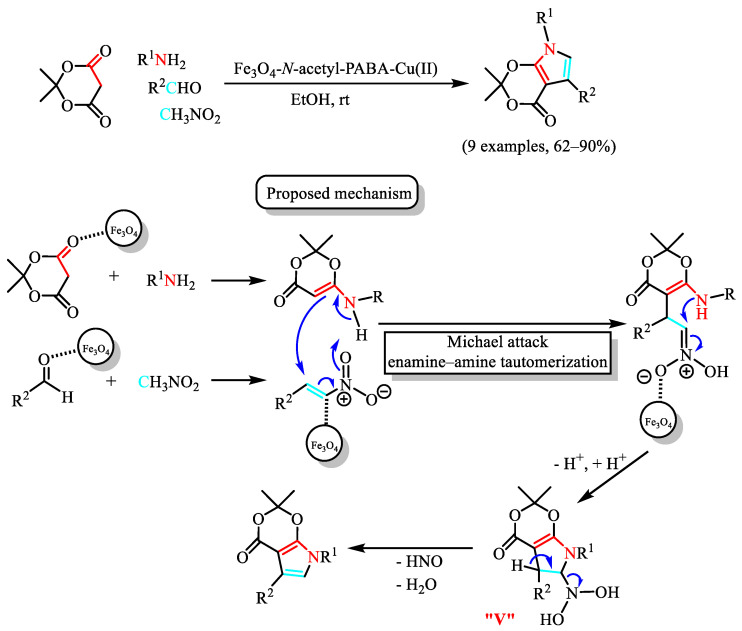
Four-component MCR synthesis of 2,2-dimethyl-2*H*-[1,3]dioxino [4,5-*b*]pyrrol-4(7*H*)-ones in the presence of Fe_3_O_4_-*N*-acetyl-PABA-Cu(II).

*N*-Acetyl-PABA@Cu(II) supported on Fe_3_O_4_ has been recently used as a new heterogeneous catalytic system to assist the MCR synthesis of 2,2-dimethyl-2*H*-[1,3]dioxino [4,5-*b*]pyrrol-4(7*H*)-ones under ultrasonic irradiation (Scheme 28) [74]. The catalyst plays a multiple role in favoring the MIRC cascade through path “***c***” of Scheme 3.

**Scheme 29:**
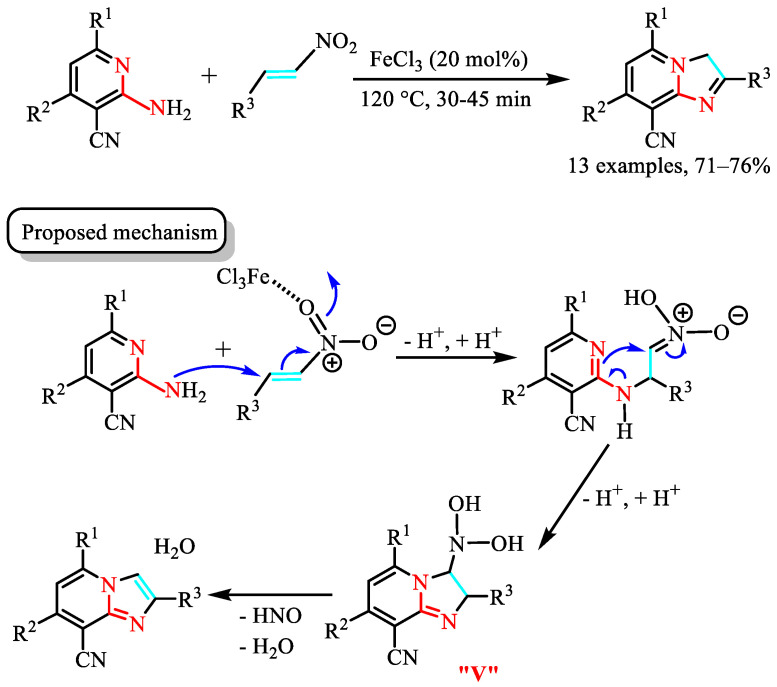
FeCl_3_-Catalyzed synthesis of 3-arylimidazo [1,2-*a*]pyridines from 2-amino pyridines and nitrostyrenes.

In order to provide a more exhaustive view of the access to five-membered *N*-heterocycles via nitroolefins acting as biselectrophiles, it is worth citing, among other similar examples reviewed elsewhere [25,75], one recent report on the assembly of a pyrrole ring fused to a pyridine ring, i.e., the synthesis of 3-arylimidazo [1,2-*a*]pyridines from 2-amino pyridines and nitrostyrenes catalyzed by FeCl_3_ (Scheme 29) [76]. The role of FeCl_3_ is to increase the electrophilicity of the nitroolefin and thus facilitate the aza-Michael attack by the 2-aminogroup of the heterocycle. The MIRC annulation is followed by aromatization of the newly formed fused heterocycle through path “***c***” of Scheme 3.

**Scheme 30:**
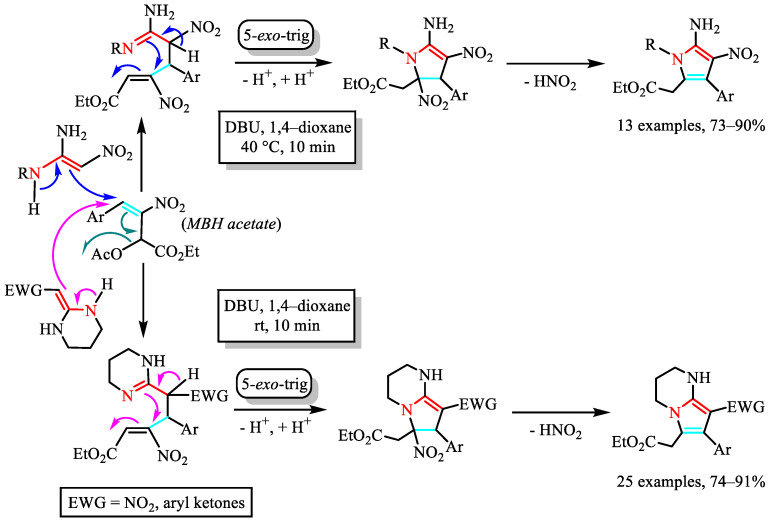
Isolated and fused 2-aminopyrroles by reaction between enediamines and MBH adducts (acetates) of nitroolefins.

In 2019 a synthesis of fully substituted isolated and fused 2-aminopyrroles by reaction between enediamines and MBH adducts (acetates) of nitroolefins was published, (Scheme 30) [77]. The products, provided with amino functionalities, are obtained in moderate to excellent yields. It is worth noting that, besides the activation by a nitro group of the olefinic double bond of the MBH acetate, a second nitro group contributes in turn to draw electrons towards the C_β_ of the enamine, thus favoring its nucleophilic attack to the C_β_ of the nitroolefin. Furthermore, the authors suggest a stabilizing effect of the enaminic nitro group on both the initial coupling product and the dihydropyrrole by means of H-bonding with the adjacent amino group.

### 2.2. Five-Membered Oxygen Heterocycles

Besides the construction of the pyrrole ring, the use of nitroolefins as α,β-biselectrophiles, as an alternative to their involvement in 1,3-dipolar cycloadditions, for the approach to obtain furans has in turn found rather widespread application in the last decade [78]. Of course, research in the field is fostered by the biological/pharmacological interest in the dihydrofuran/furan nucleus, either isolated or fused in more complex heterocyclic structures [78,79].

A consistent number of reports in the field concern *gem*-halonitroolefins, in particular *gem*-bromonitroolefins [13,14,80], which offer the advantage of providing at C_α_ a far better leaving group than the nitro group, thus facilitating the heteroring-closing step via an intramolecular S_N_2 nucleophilic displacement, within path “***b***” of Scheme 3 [78].

#### 2.2.1. Isolated Furan Rings

**Scheme 31:**
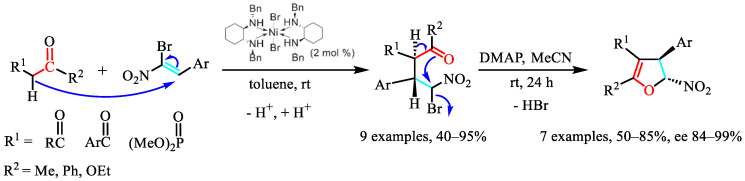
*gem*-Bromonitroolefins as building blocks in the synthesis of 4,5-dihydrofurans via chiral precursors and dimethylaminopyridine-assisted ring closure.

As regards isolated furan rings, *gem*-bromonitroalkenes have been used as building blocks for the assembly of 2-nitro-*trans*-2,3-dihydrofurans by coupling with different active-methylene compounds such as β-diketones, keto esters, and keto phosphonates in the presence of a chiral Ni(II) catalyst [81] (Scheme 31): the isolable Michael addition products could be cyclized via intramolecular S_N_2 nucleophilic displacement in the presence of dimethylaminopyridine (DMAP) [81] or NaOH [79].

**Scheme 32:**
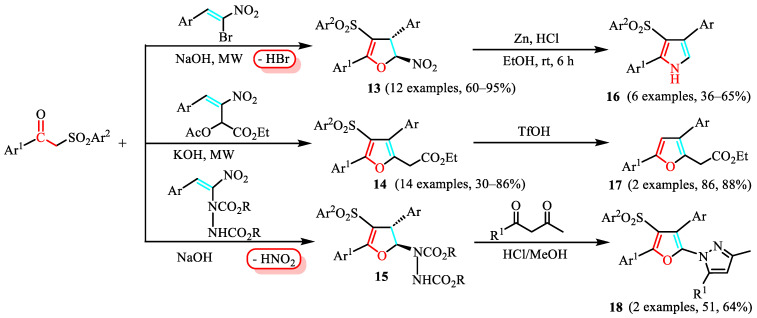
Polysubstituted furans or dihydrofurans from β-keto sulfones and different nitroolefins.

β-Keto sulfones have been reported as effective three-atom partners for different nitroolefins or their MBH adducts for the assembly of dihydrofurans or furans, amenable to further manipulation (Scheme 32) [79].

The formation of **13** with the elimination of HBr is straightforward (cf. Scheme 31, path “***b***” of Scheme 3): interestingly enough, the nitro group reduction eventually leads to polysubstituted pyrroles.

**Scheme 33:**
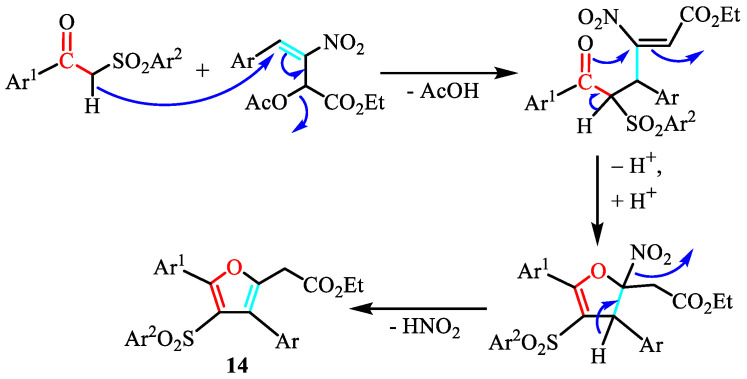
Proposed mechanism for the formation of furans (**14**) (cf. Scheme 27).

**Scheme 34:**
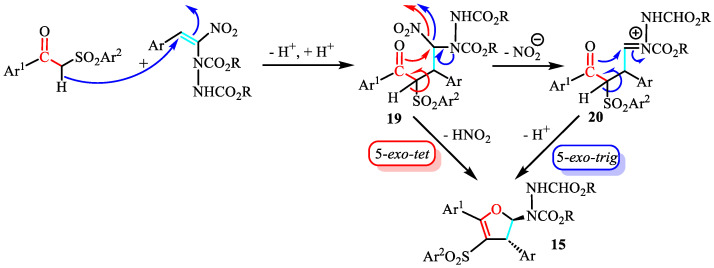
Proposed alternative, possibly concurrent mechanisms for the formation of furans (**15**).

As far as the use of MBH adducts of nitroolefins is concerned, specific mechanisms for the formation of **14** (which can be eventually desulfonylated) (Scheme 33) and of **15** (amenable in turn to further elaboration) (Scheme 34) have been proposed [79]. In the former case, with the participation of MBH adduct **A**, the mechanism is the one already reported in Scheme 27. In the latter case, with the participation of MBH adduct **B**, both a 5-*exo*-tet (red arrows from the intermediate **19**) and a 5-*exo*-trig ring process (blue arrows from **20**) may concur in the assembly of the heteroring with 100% diastereoselectivity: in either case, the ring-closing step depicts a nucleophilic attack to C_α_ of the initial nitroolefin, and it should also be remarked that the 5-*exo*-tet pathway is an example of path “***a***” of Scheme 3, with the elimination of nitrite ion along with ring closure.

**Scheme 35:**
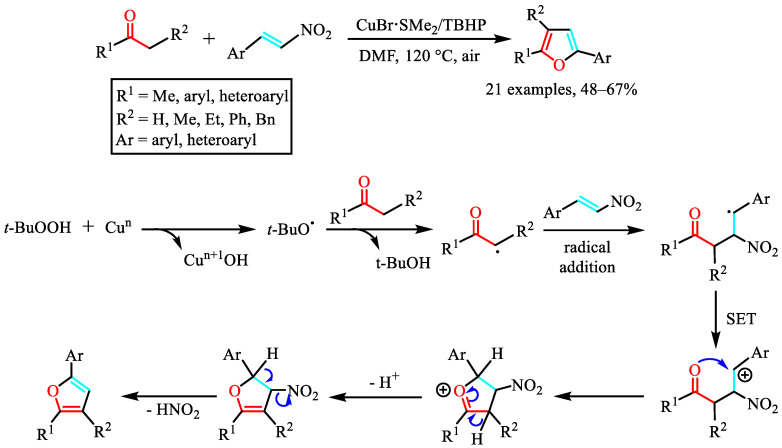
Scope and proposed partially radical mechanism for the formation of polysubstituted furans from ketones and nitrostyrenes assisted by CuBr.SMe_2_.

An interesting synthesis of polysubstituted furans from nitroolefins and ketones was carried out in DMF in the presence of Cu salts and *tert*-butyl hydroperoxide (TBHP). The proposed mechanism features an initial radical coupling between a β-carbonyl radical and the C_α_ of the nitroolefin, followed by a Single-Electron Transfer (SET) and ring closure by nucleophilic attack at C_β_ (Scheme 35) [82]. The inhibitory effect of added radical scavengers in control experiments clearly sustains the participation of radical species.

#### 2.2.2. Fused Furan Rings

**Scheme 36:**
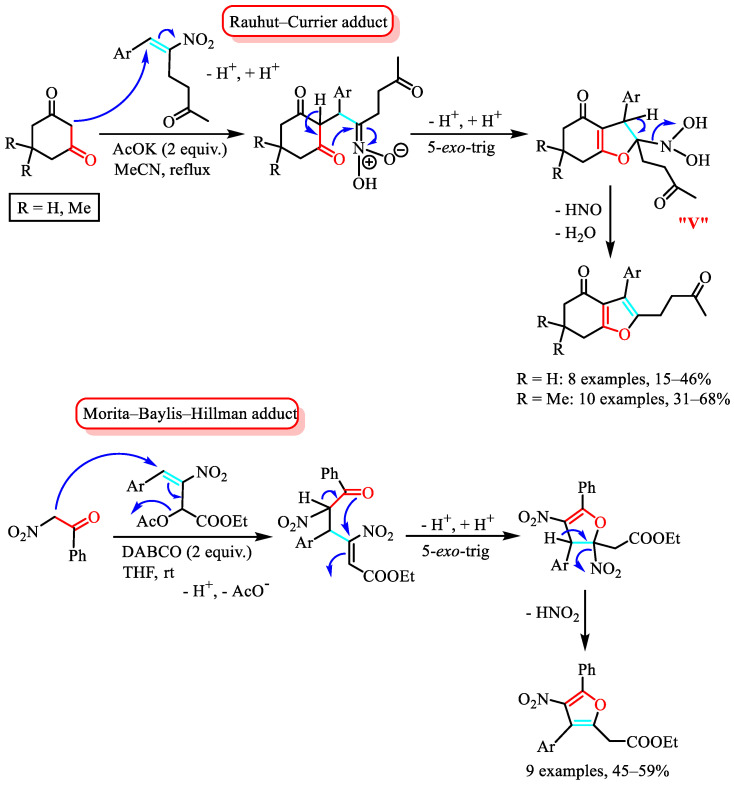
Furans and furan-fused heterocycles from carbonyl or 1,3 dicarbonyl starting materials by exploiting the twofold electrophilic reactivity of RC or MBH adducts of nitroolefins of type **A** (cf. Scheme 32).

Furans and furan-fused heterocycles of potential biological activity were regioselectively synthesized from carbonyl or 1,3-dicarbonyl starting materials by exploiting the twofold electrophilic reactivity of Rauhut–Currier (RC) (through path “***c***” of Scheme 3) or MBH adducts of nitroolefins (Scheme 36) [43,83]. Interestingly enough, in the second case reported in the scheme the nitro-group activates both reagents for the coupling.

**Scheme 37:**
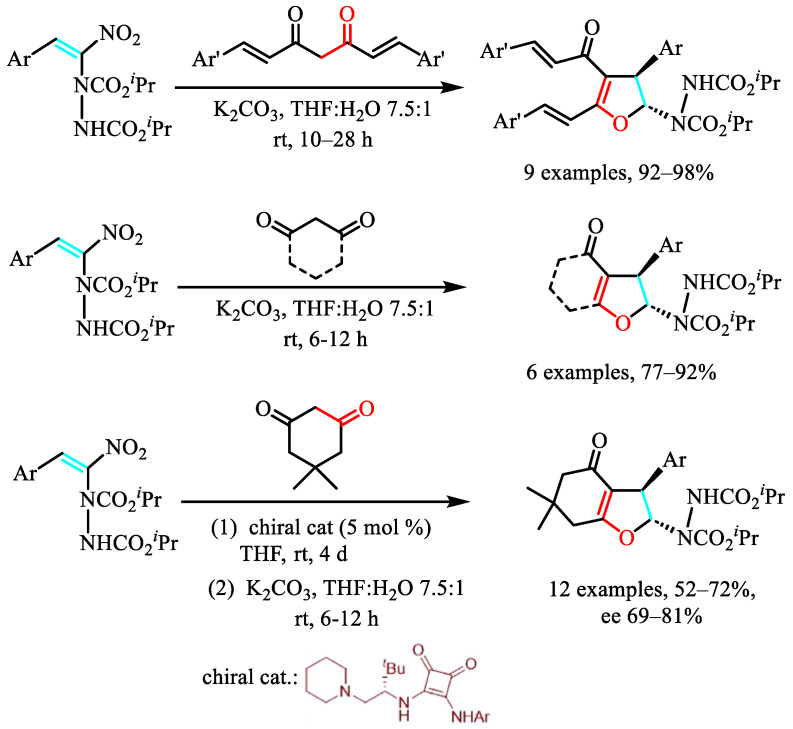
Diastereoselective or enantioselective construction of isolated or fused dihydrofuran rings by reaction of 1,3-dicarbonyls with MBH adducts of nitroolefins of type **B** (cf. Scheme 32).

MBH adducts of nitroolefins have been also employed for a diastereoselective synthesis of isolated or fused dihydrofuran rings by reaction with 1,3-dicarbonyls (Scheme 37) [84]. In each case the proposed mechanism is that reported in Scheme 34 for a similar MBH adduct with a 5 *exo*-trig ring closure.

In one case, the use of a chiral catalyst produced enantioselectivity.

**Scheme 38:**
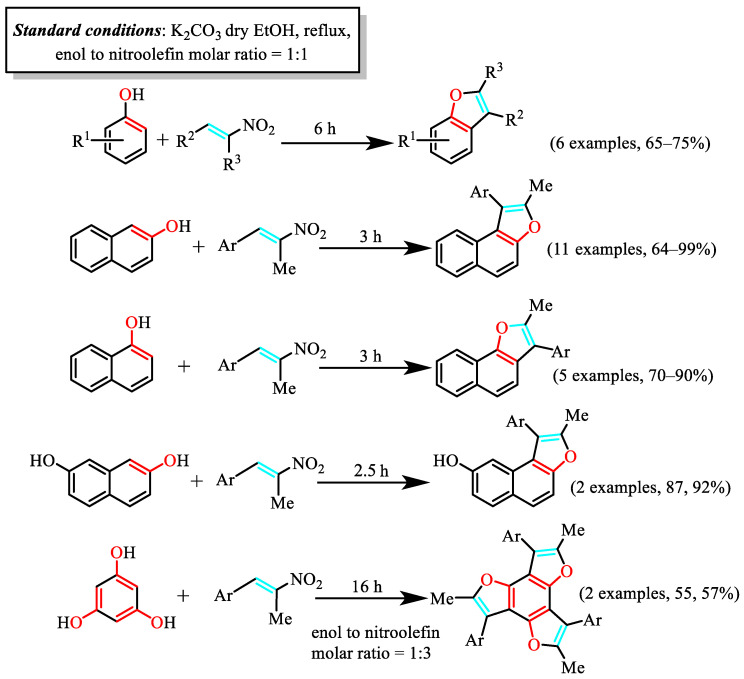

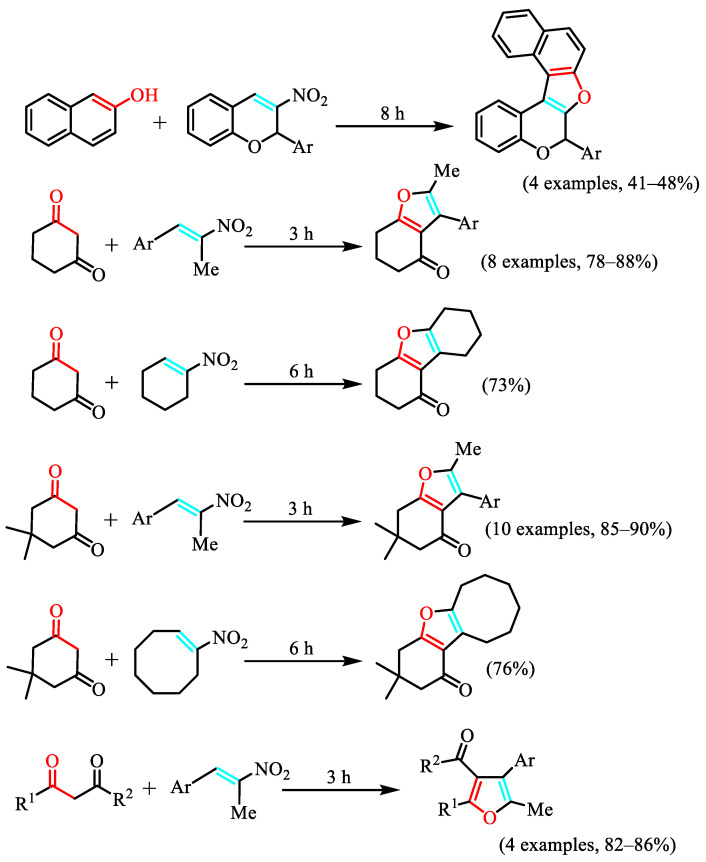
Synthesis of various furan-fused heterocycles from aliphatic and aromatic enols in EtOH/K_2_CO_3_.

In prosecution of previous research in the field, phenols, naphthols and 1,3-dicarbonyls had been used for the assembly of furo-fused heterocycles in EtOH, in the presence of K_2_CO_3_ (Scheme 38) [85]. The reaction mechanism follows path “***c***” of Scheme 3, previous enolization of the carbonyl compounds.

**Scheme 39:**
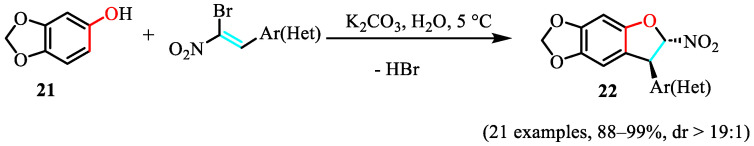
Highly efficient and scalable synthesis of *trans*-dihydrodioxolobenzofurans in environmentally friendly conditions.

*trans*-Benzofuran derivatives **22** were obtained in high yields (also on a gram scale) by coupling of *gem*-bromonitrostyrenes with phenol **21** in environmentally friendly conditions (Scheme 39) [86].

**Scheme 40:**
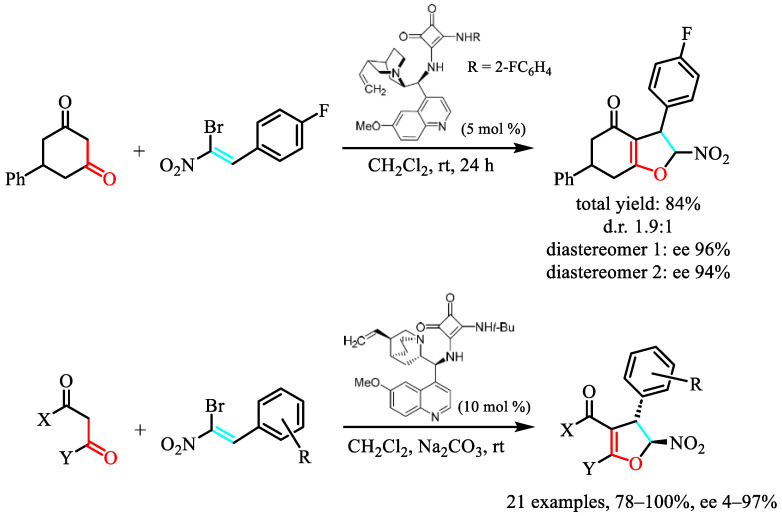
Polysubstituted dihydrofurans from β-diketones or β-ketoesters and *gem*-bromonitroolefins [87,88].

*gem*-Bromonitroalkenes also proved to be effective precursors of dihydrofurans by coupling with β-diketones or β-ketoesters under chiral catalysis in the presence of Na_2_CO_3_ (Scheme 40) [87,88].

According to the mechanistic model provided by the authors [88], the nucleophile is activated by H-bonding with the quinuclidine moiety of the organocatalyst; simultaneously, the electrophile is activated by double H-bonding of the nitro group with the squaramide hydrogens.

**Scheme 41:**
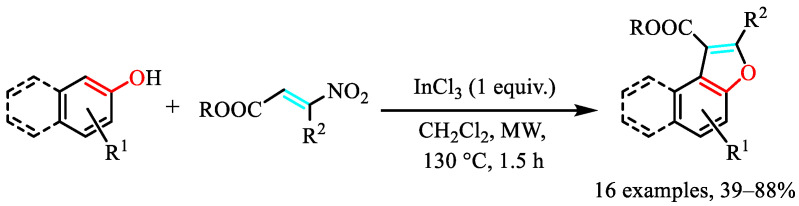
Benzo- and naphthofurans from phenols or naphthols and β-nitroacrylates.

Strongly electrophilic β-nitroacrylates have very recently proven excellent precursors of benzofurans or naphtho [2,1-*b*]furans in a Lewis acid-catalyzed, MW-assisted MIRC process which overcomes drawbacks such as complex starting materials, expensive catalysts, harsh reaction conditions and long reaction times (Scheme 41) [89].

**Scheme 42:**
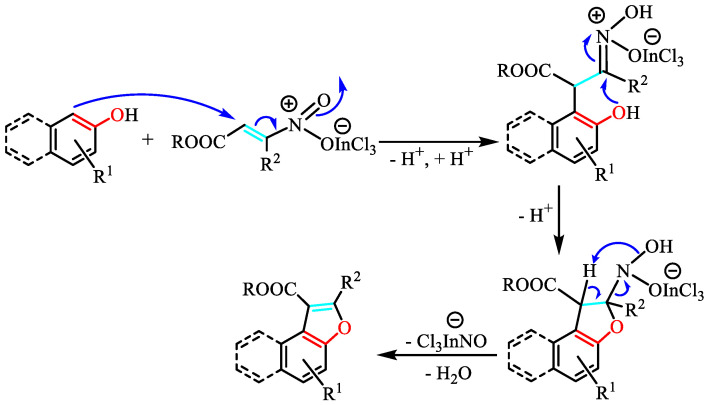
Mechanistic proposal for the synthesis of benzo- and naphthofurans from phenols or naphthols and β-nitroacrylates.

The proposed mechanism is a MIRC process, characterized by LA bonding to the nitro group, following path “***c***” of Scheme 3 (Scheme 42).

**Scheme 43:**
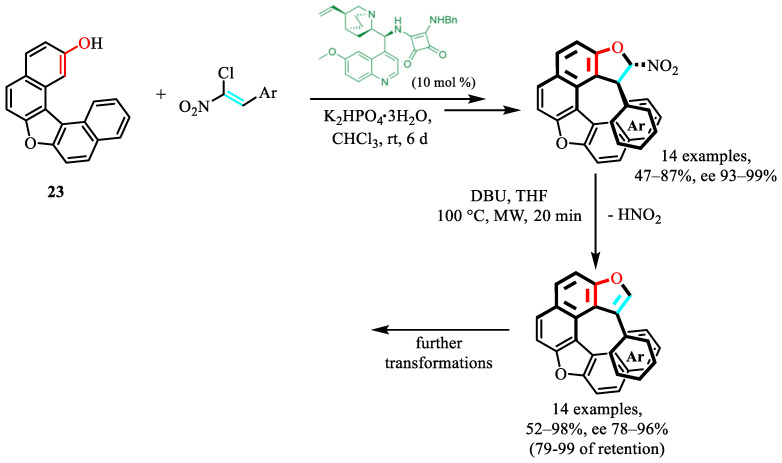
Dioxa[6]helicenes formed from **23** and *gem*-chloronitroolefins.

Heterohelicenes are *ortho*-fused heteropolycyclic aromatic compounds which, thanks to a stable screw-shaped conformation, have recently been revealed to be promising scaffolds for developments in many fields [90]. An expedient synthesis of a new family of configurationally stable dioxa[6]helicenes was realized by coupling of β-naphthol **23** with *gem*-chloronitroolefins: the so-formed *trans*-dihydrofurans successively underwent aromatization via MW-assisted HNO_2_ elimination in the presence of DBU with high enantiopurity (Scheme 43) [90].

**Scheme 44:**
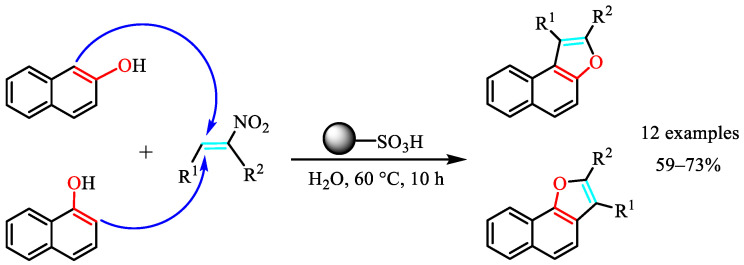
Sulfonic acid-catalyzed synthesis of naphthofurans from naphthols and nitroolefins.

The protocol cited above for the synthesis of benzoindoles (Section 2.1.2, Scheme 20 and Scheme 21) has also been applied as an efficient approach to obtain naphthofurans (Scheme 44) [67].

**Scheme 45:**
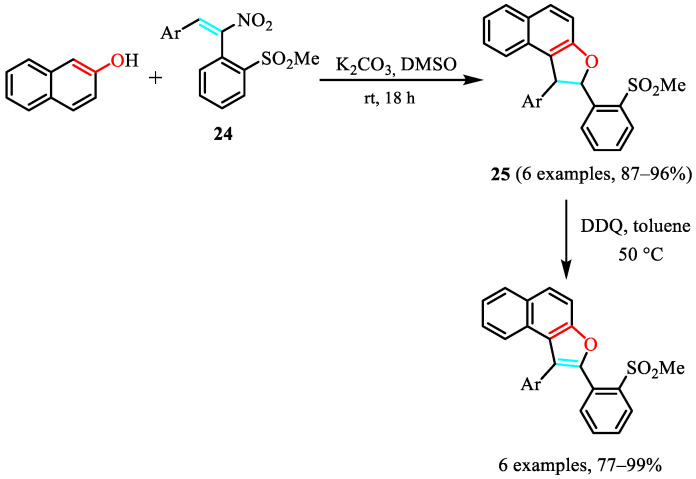
Naphthodihydrofurans **25** and naphthofurans from β-naphthol and nitrostilbenes **24**.

The nitrostilbenes **24** have recently proven to be excellent precursors of naphthodihydrofurans **25**, which have been easily and effectively aromatized with DDQ in toluene (Scheme 45) [91].

**Scheme 46:**
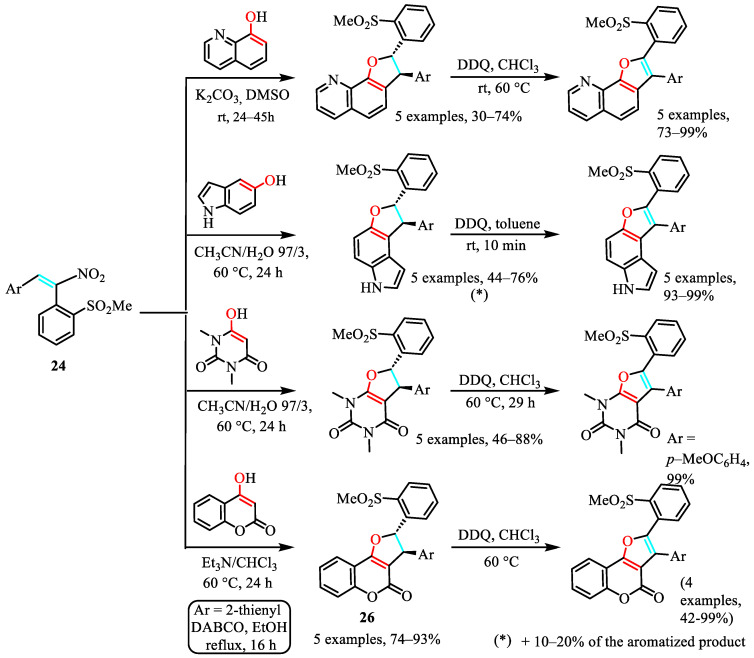
Heteropolycycles formed from the initial coupling of aromatic enols and nitrostilbenes.

More recently, the same authors have successfully extended the use of nitrostilbenes (**24**) to the synthesis of different fused heteropolycycles (Scheme 46) [92]. The fused dihydrofuran derivatives from the annulation (among which also the furo [3,2-*c*]coumarins (**26**) are of particular potential biological interest: see the paragraph dedicated to such fused heterocycles hereinafter), showing complete diastereoselectivity, were aromatized with DDQ/toluene before being subjected to further transformations leading to more complex, completely fused heteropolycycles.

**Scheme 47:**
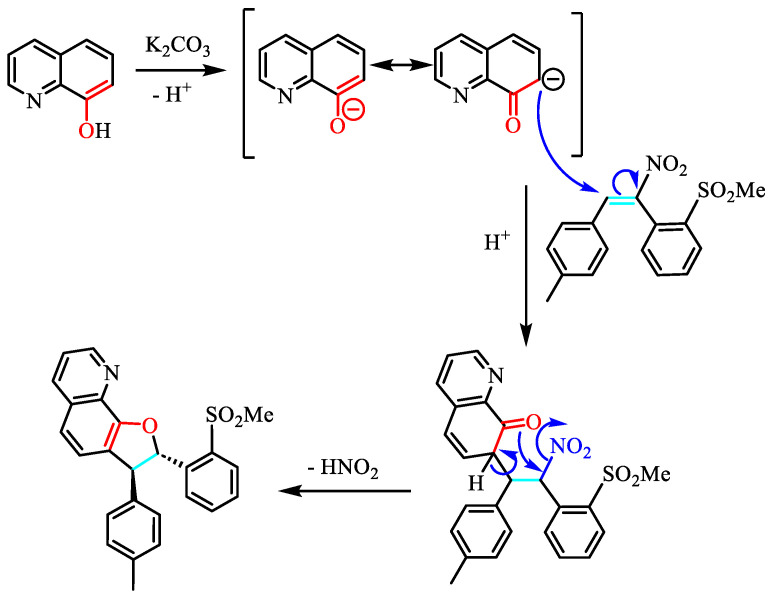
Proposed mechanism for the dihydrofuran-ring assembly of Scheme 45 and Scheme 46.

The dihydrofuran-ring assembly of Scheme 45 and Scheme 46 has been rationalized on the grounds of a MIRC process following path “***a***” of Scheme 3 (Scheme 47, where the mechanism is sketched for the model 8-hydroxyquinoline) [92]. The structure of the isolated dihydro-derivatives clearly speaks for a nitro group substitution within a S_N_2 intramolecular ring-closing process, being obviously incompatible with path “***c***” of Scheme 3.

**Scheme 48:**
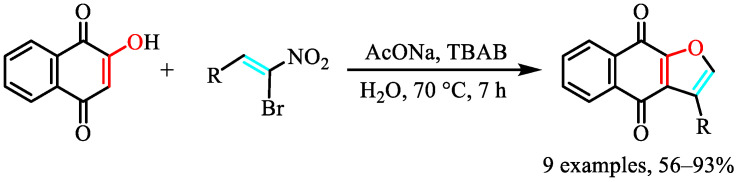
Synthesis of furanonaphthoquinone from 2-hydroxynaphthoquinone and *gem*-bromonitroalkenes.

As already cited (see Scheme 27), quinonoid naphthofurans of potential pharmacological activity were synthesized from *gem*-bromonitroalkenes and 2-hydroxynaphthalene-1,4-dione in the presence of AcONa and tetrabutylammonium bromide (TBAB) (Scheme 48) [72,73].

**Scheme 49:**
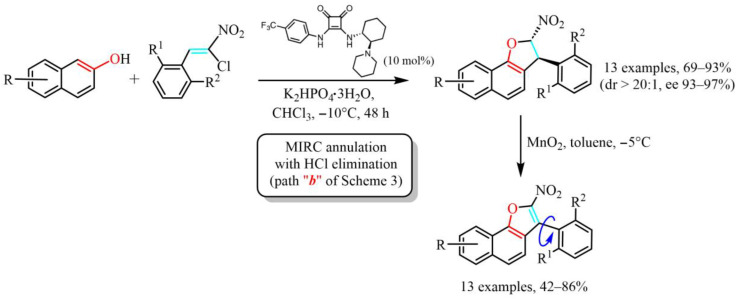
MIRC construction of hindered *trans*-dihydrofuran-fused polycycles from *gem*-chloronitroolefins and β-naphthols and successive aromatization [93].

**Scheme 50:**
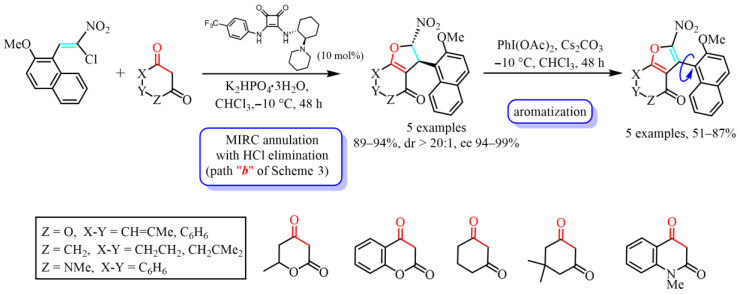
MIRC construction of hindered *trans*-dihydrofuran-fused polycycles from *gem*-chloronitroolefins and β-dicarbonyls and successive aromatization [93].

In the context of atropisomerism studies, *trans*-dihydrofuran-fused polycycles were generated from *gem*-chloronitroolefins and β-naphthols (Scheme 49) or cyclic β-dicarbonyls (Scheme 50) in the presence of a chiral catalyst via an enantioselective organocatalyzed 1,4-addition followed by an intramolecular diastereoselective O-alkylation (i.e., a MIRC annulation). The *trans*-dihydrofurans were then aromatized by oxidation to the final desired furans [93].

**Scheme 51:**
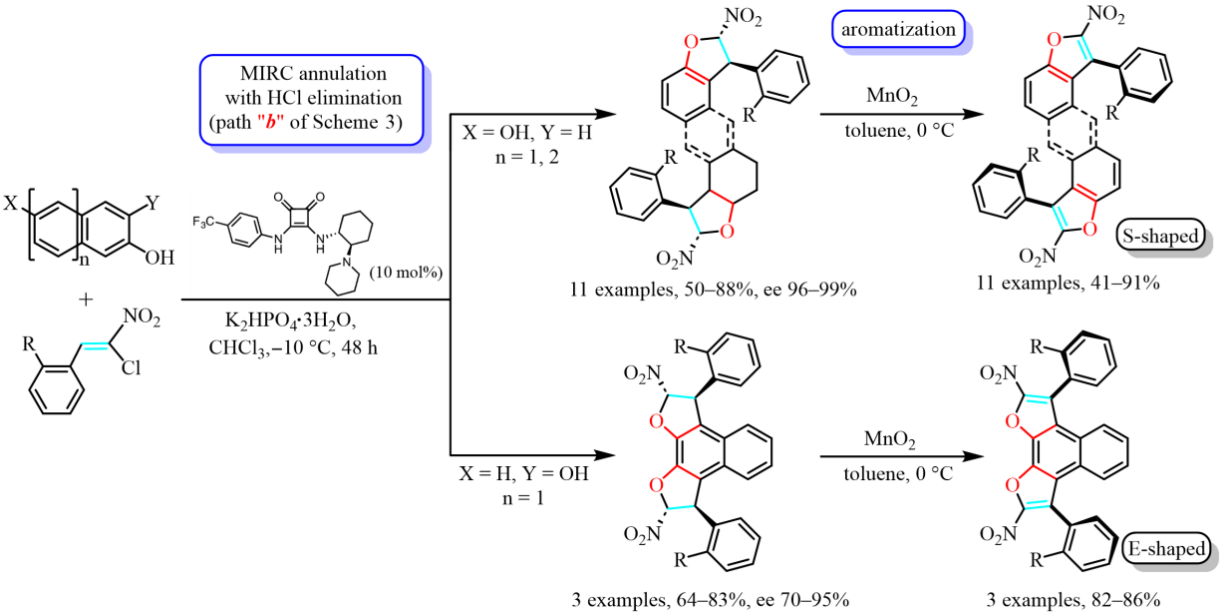
MIRC annulation followed by aromatization for the construction of S- and E-shaped bis-benzofuran atropisomeric oligoarenes from *gem*-chloronitroolefins and aromatic diols.

Aromatic diols were also used, under conditions analogous to those of the previous report, for the assembly of complex structures such as dinitro-*trans*-dihydrodifurans and dinitrodifurans provided with two distal C–C stereogenic axes (Scheme 51) [94].

**Scheme 52:**
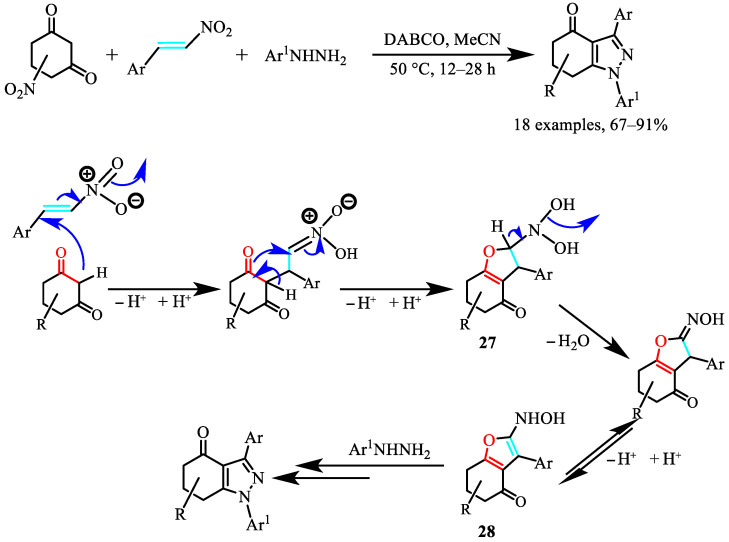
Three-component regioselective synthesis of tetrahydroindazolones through furo-fused intermediates.

The three-component reaction of nitrostyrenes with 1,3-cyclohexanediones and arylhydrazines of Scheme 52 has been reported to lead to tetrahydroindazolones through furo-fused intermediates [95].

Interestingly, the intermediate **27** (corresponding to **V** of Scheme 3) aromatizes to **28** through elimination of H_2_O and tautomerization of the resulting oxime, thus maintaining nitrogen functionality while undergoing hydrazine addition, instead of eliminating both H_2_O and HNO, as usually reported as completion of the assembly of the five-membered heteroring.

##### Furocoumarins (Furan-Fused Chromen-4-Ones)

Among different fused heterocycles characterized by the coumarin core, the special role played in particular by furocoumarins (furan-fused chromen-4-ones), and more specifically by furo [3,2-*c*]coumarins in chemistry, biochemistry, and pharmacology [96,97], is testified to by the number of synthetic approaches which continue to appear in the literature [98]: a recent paper reports a list of the main different protocols for the assembly of furo [3,2-*c*]chromen-4-ones from 4-hydroxycoumarins in particular [99]. In this field, among a number of two-carbon electrophilic partners, nitroolefins surely play a prominent role as building blocks for the assembly of the fused furan ring.

**Scheme 53:**
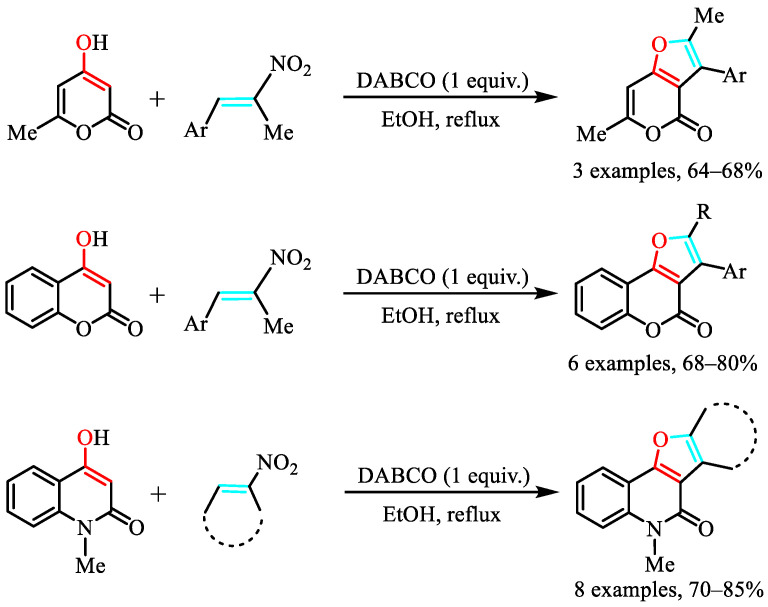
DABCO-catalyzed synthesis of furo-fused heterocycles from aromatic enols.

A simple, low-cost, one-pot method for the synthesis of furocoumarins and furoquinolinones was developed by coupling aromatic enols with nitrostyrenes under DABCO catalysis (Scheme 53) [82].

**Scheme 54:**
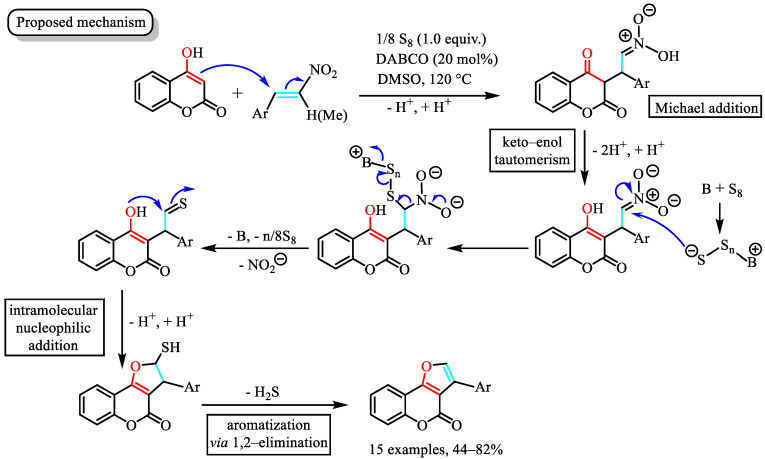
Metal-free, sulfur-promoted synthesis of furocoumarins from 4-hydroxycoumarin and nitrostyrenes.

A DABCO-catalyzed, sulfur-assisted, metal-free strategy has been very recently applied for the synthesis of furocoumarins from 4-hydroxycoumarin and nitrostyrenes (Scheme 54) [100]. It is interesting that, although the nitrostyrene behaves as a biselectrophile, according to the proposed mechanism, C_α_ undergoes intermolecular attack by the catalyst and the furan-ring closure occurs when the nitro functionality has already been lost.

**Scheme 55:**
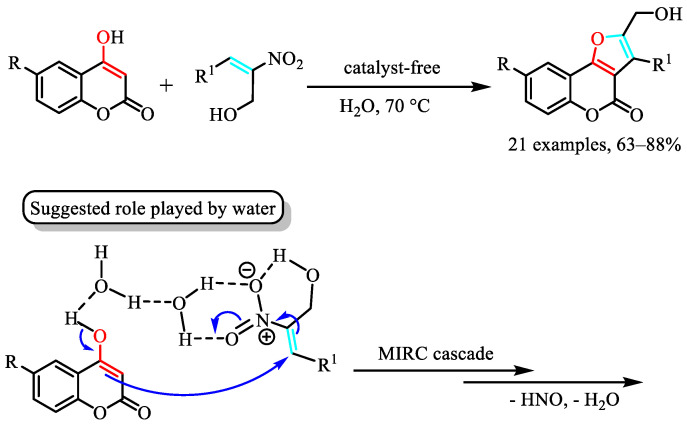
Catalyst-free, water assisted MIRC coupling to furo [3,2-*c*]chromen-4-ones.

A straightforward, catalyst-free access to furo [3,2-*c*]chromen-4-ones was realized by means of the coupling between 4-hydroxycoumarins and (*E*)-3-substituted-2-nitro-2-propenols thanks to the participation of water molecules to favor the initial Michael addition which leads to the final product through path “***c***” of Scheme 3 (Scheme 55) [101].

**Scheme 56:**
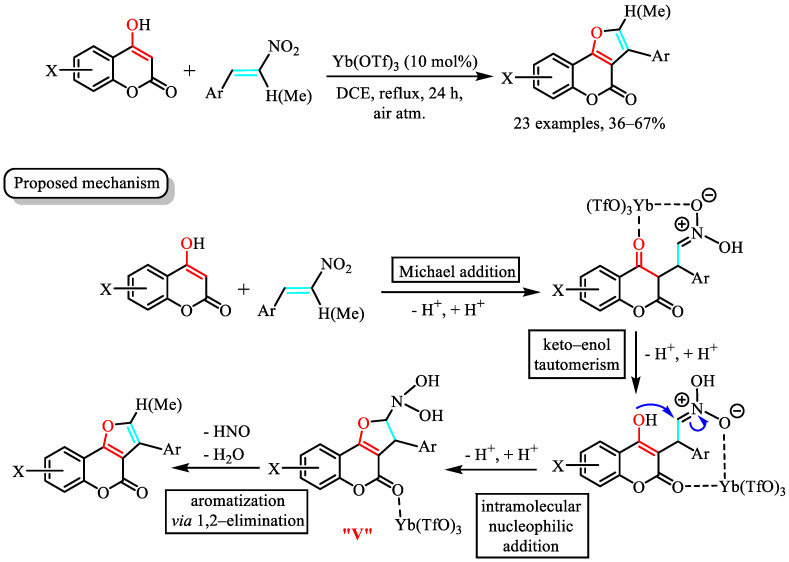
Lewis acid-catalyzed synthesis of furocoumarins from 4-hydroxycoumarins and nitrostyrenes.

A wide-scope synthesis of furo [3,2-*c*]chromen-4-ones from 4-hydroxycoumarins and nitrostyrenes can be achieved, with satisfactory yields, by Yb(OTf)_3_ catalysis. The proposed mechanism is path “***c***” of Scheme 3 (Scheme 56) [99]. The proposed mechanism is a MIRC annulation, following path “***c***” of Scheme 3, with eventual aromatization via elimination of HNO and H_2_O.

**Scheme 57:**
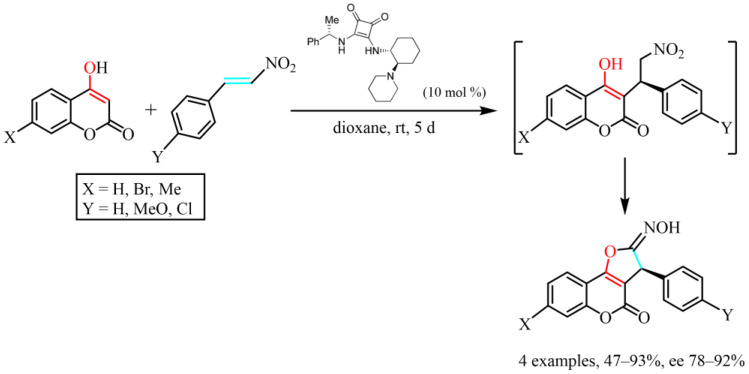
Enantioselective MIRC approach to synthesis of dihydrofurocoumarins from 4-hydroxycoumarins and β-nitrostyrenes.

An enantioselective synthesis of dihydrofurocoumarins by coupling of 4-hydroxycoumarins with β-nitrostyrenes in the presence of a squaramide chiral catalyst was reported (Scheme 57) [102]. As to the mechanism, most likely the “aci” tautomer of the proposed intermediate undergoes ring closure via intramolecular nucleophilic addition, followed by water elimination to the final oxime.

**Scheme 58:**
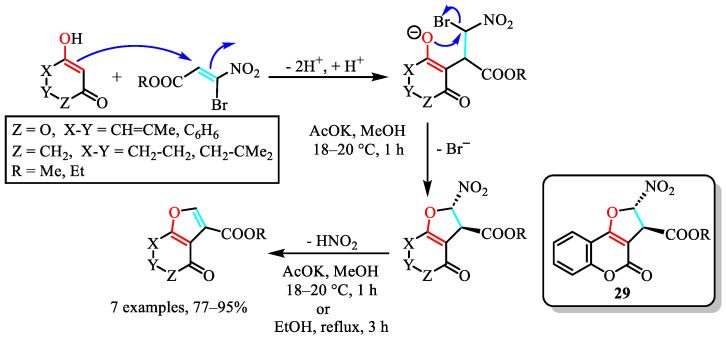
MIRC approach to furo-fused heterocycles from *gem*-bromonitroacrylates with bromide ion intramolecular substitution.

In *gem*-bromonitroacrylates the electrophilicity of nitroolefins is increased significantly by summing up the effect of both the ester group at C_β_ and the halogen at C_α_. Such building blocks were applied in the synthesis of a number of furo-fused heterocycles in the presence of AcOK (Scheme 58) [103,104]. In the reaction with 4-hydroxycoumarin the use of an equimolar amount of base allowed isolation of the intermediate dihydrofurocoumarin (**29**), confirming the proposed mechanism via path “***b***” of Scheme 3 [103].

**Scheme 59:**
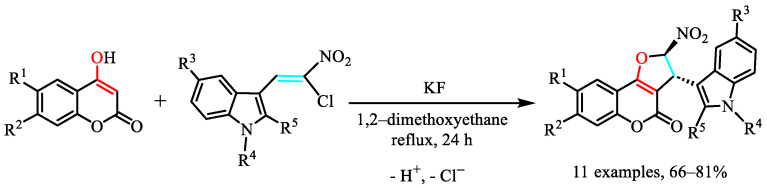
MIRC construction of indolyl-substituted racemic dihydrofurocoumarins.

Condensed dihydrofurocoumarins with antimicrobial activity were obtained from 4- hydroxycoumarins and a *gem*-chloronitroolefin carrying an indolyl group at C_α_ in the presence of KF (Scheme 59) [105].

**Scheme 60:**
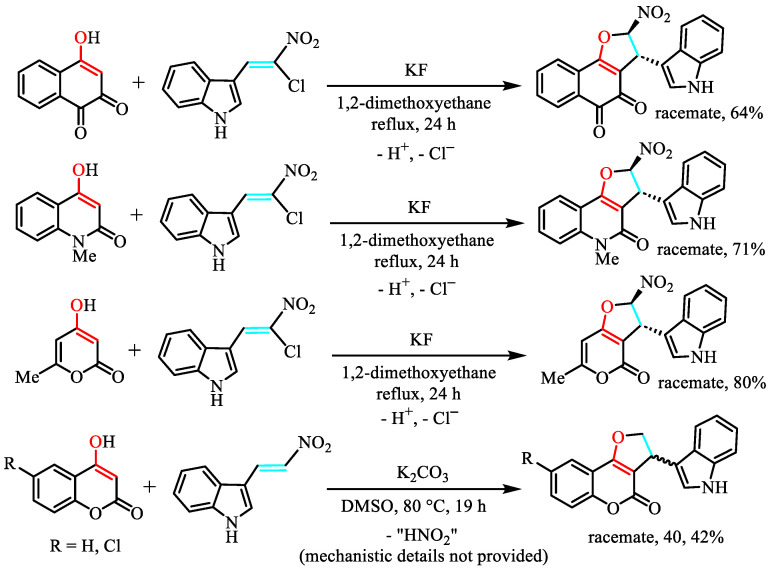
MIRC construction of indolyl unsubstituted dihydrofuro- or furo-fused aromatics or heteroaromatics.

In search of related structures with potentially similar or better activity, the synthesis was extended to the coupling between (*a*) analogues of 4-hydroxycoumarin with unsubstituted 1-chloro-1-nitro-2-(3-indolyl)ethylene and (*b*) 4-hydroxycoumarins and unsubstituted 1-(3-indolyl)-2-nitroethylene, in DMSO in the presence of K_2_CO_3_ (Scheme 60).

### 2.3. Five-Membered Sulfur Heterocycles

**Scheme 61:**
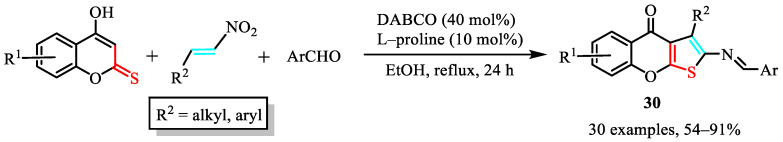
Substituted thieno [2,3-*b*]chromen-4-ones **30** via a base-promoted, three-component reaction.

In the rather limited panorama relevant to the use of nitroolefins for the synthesis of pentatomic *S*-heterocycles, thieno [2,3-*b*]chromen-4-ones **30** have been regioselectively synthesized through a three-component MCR from 4-hydroxythiocoumarins, aryl aldehydes and *trans*-β-nitrostyrenes (Scheme 61) [106].

**Scheme 62:**
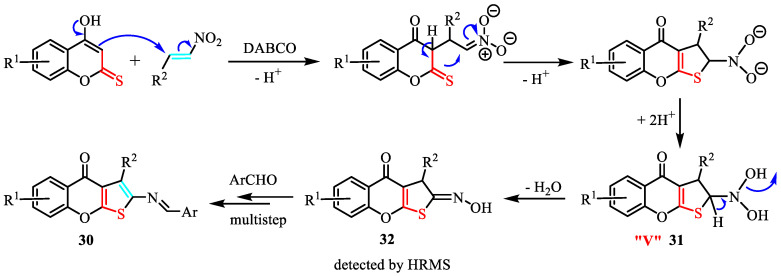
Proposed mechanism for the formation of dihydro- (**32**) or thieno [2,3-*b*]chromen-4-ones (**30**).

As proposed by the authors, the assembly of the fused polyheterocycle (**32**, detected by HRMS but not isolated) occurs via path “***c***” of Scheme 3 (Scheme 62) and is followed by the “one-pot” functionalization with the arylimino moiety.

From a mechanistic point of view, it is worth noting that (*a*) the HRMS detection of **32** could be regarded as evidence of the intermediacy of **31**, i.e., the intermediate V of path “***c***” of Scheme 3, although (*b*) interestingly enough, as already pointed out regarding Scheme 52, **31** does not aromatize eliminating HNO and H_2_O, as it is usually reported for suggested analogous intermediates.

**Scheme 63:**
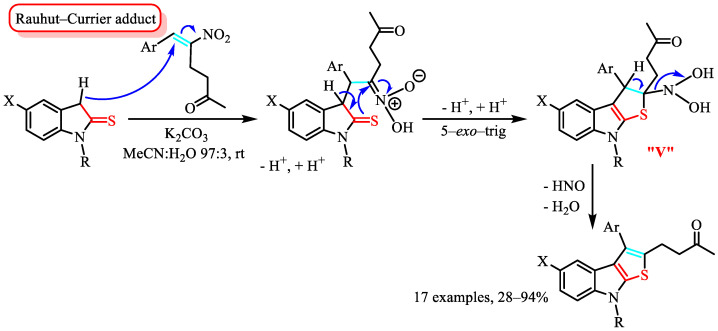
Functionalized thieno [2,3-*b*]indoles via base-mediated [3+2]-annulation of indoline-2-thiones with RC adducts of nitroalkenes.

**Scheme 64:**
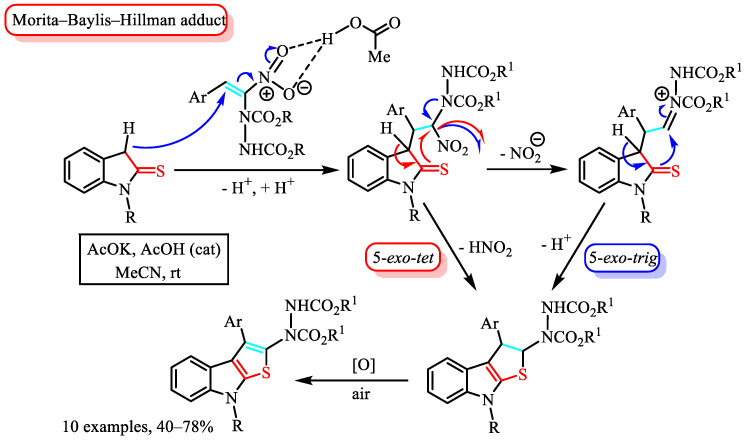
Functionalized thieno [2,3-*b*]indoles obtained via base-mediated [3+2]-annulation of indoline-2-thiones with Morita−Baylis−Hillman adducts of nitroalkenes.

In 2018, the same research group that published what was reported in Scheme 36, above [83], reported a straightforward protocol for the synthesis of functionalized thieno [2,3-*b*]indoles by base-mediated [3+2]-annulation of indoline-2-thione with RC (Scheme 63) or MBH adducts of nitroalkenes [43] (Scheme 64), gratified by complete regioselectivity, broad substrate scope, and mild reaction conditions [107]. Moreover, thanks to the functionalities present, the obtained thieno [2,3-*b*]indoles are amenable to further synthetic elaboration.

## 3. Conclusions

The nitro group is one of the most useful functionalities in organic synthesis for the assembly of intermediates or final products of relevance in many fields of organic chemistry.

The present review offers an update in the specific field of the employment of conjugated nitroolefins as powerful starting materials for the construction of heterocycles. Although limited to the intervention of such building-blocks as 1,2-biselectrophilic, two-atom partners for the assembly of five-membered monoheterocycles, the number and relevance of the latest literature reports cited herein are clear evidence of the continuous interest in the field, in particular regarding the nitroolefin-based construction of five-membered *N*-heterocycles, a subject to which a few reviews have been recently devoted.

This review also intends to provide a full panorama of the mechanistic hypotheses advanced by the researchers, actually showing that a number of different paths have been supposed to be operative, depending on the structure of the reagents, and mainly that of the nitroolefin itself, within a common MIRC process. From this point of view, significant variants have been provided by the use of MBH adducts of the nitroolefin. Sometimes, mechanistic proofs can be achieved by means of the isolation or instrumental identification of intermediates, although most often hypotheses align with proposals well consolidated in the literature.

Driven by the search for optimization of reaction yields and scope, as well as practical simplicity and environmental sustainability, there is great variability as far as experimental conditions are concerned within the field, for instance, in terms of the utilization of homogeneous, heterogeneous or organo-metal catalysis; avoidance of heavy-metal catalysts or, at least, if they are necessary, their reusability being ensured; and the use of nanoparticles, microwave irradiation, liquid-assisted grinding, etc. Chiral catalysis is in turn exploited, when necessary, to reach the high level of enantioselectivity of dihydroheterocycles.

Finally, operatively, a good number of approaches take advantage of the MCR protocol, which is based on the use of simpler reagents in one-pot processes, avoiding the isolation of intermediates, and is characterized by high atom economy and yields.

## Data Availability

No new data were created or analyzed in this study. Data sharing is not applicable to this article.

## Abbreviations

The following abbreviations are used in this manuscript:

DABCO	1,4-DiAzaBiCyclo [2.2.2] Octane
DBU	1,8-DiazaBicyclo [5.4.0] Undec-7-ene
DDQ	2,3-Dichloro-5,6-Dicyano-1,4-benzoQuinone
DES	Deep Eutectic Solvent
DMAP	DiMethylAminoPyridine
DMSO	DiMethylSulfOxide
LAG	Liquid-Assisted Grinding
MBH	Morita–Baylis–Hillman
MCR	Multicomponent Reaction
MIL	Material from Institute Lavoisier
MIRC	Michael-Initiated Ring Closure
MOF	Metal–Organic Framework
MW	MicroWave
NMPH	*N*-Methyl-2-Pyrrolidonium
NP	Nanoparticle
PABA	*Para*-AminoBenzoic Acid
PEG	PolyEthylene Glycol
RC	Rauhut–Currier
SET	Single-Electron Transfer
TBAB	TetraButylAmmonium Bromide
TBHP	*Tert*-Butyl HydroPeroxide
